# Relationship between Physical Health, Disability, and Geriatric Syndromes and Depressive Symptoms in Older Adults

**DOI:** 10.1093/geroni/igaf122.3075

**Published:** 2025-12-31

**Authors:** Poshan Dahal

**Affiliations:** Case Western Reserve University, Cleveland, Ohio, United States

## Abstract

Depression in older adults is associated with various negative health consequences. Combination of chronic conditions, functional limitations and geriatric syndromes have the potential to cause detrimental physical and psychological health outcomes. This paper aims to identify the combinations of chronic conditions, functional limitations, and geriatric syndromes that are associated with depressive symptoms among older adults. We used data from 2016-2018 Health and Retirement Study (HRS) in this study. The HRS provides a representative sample of adults aged 50 years and above in the US. We used Classification and Regression Tree Analysis in R program and calculated descriptive statistics in STATA program. Sample size of this study is 15,957. Our outcome variable was depressive symptoms. Key independent variables were chronic conditions, functional limitations, and geriatric syndromes. Other covariates included in the analysis were age, sex, marital status, income, race, and education. Fifteen percent of respondents reported depressive symptoms at 2 years. The top combinations of variables identified through the classification and regression tree analysis for depressive symptoms included having IADL limitations, being troubled with pain, and having persistent dizziness and lightheadedness (>40%). Among older adults, functional limitations and geriatric syndrome were significantly associated with psychological wellbeing than chronic conditions. This finding underscores the clinical and programmatic importance of functional limitations and geriatric syndromes in understanding and addressing the health need of older adults.

